# Carbon Nanotubes in Cancer Therapy and Drug Delivery

**DOI:** 10.1155/2012/837327

**Published:** 2011-10-18

**Authors:** Abdelbary M. A. Elhissi, Waqar Ahmed, Israr Ul Hassan, Vinod. R. Dhanak, Antony D'Emanuele

**Affiliations:** ^1^Institute of Nanotechnology and Bioengineering, School of Pharmacy and Biomedical Sciences, University of Central Lancashire, Preston PR1 2HE, UK; ^2^Institute of Nanotechnology and Bioengineering, School of Computing, Engineering and Physical Sciences, University of Central Lancashire, Preston PR1 2HE, UK; ^3^Mathematics and Sciences Unit, College of Art and Applied Sciences, Dhofar University, P.O. Box 2509, 211 Salalah, Oman; ^4^Department of Physics, University of Liverpool, Liverpool L69 3BX, UK

## Abstract

Carbon nanotubes (CNTs) have been introduced recently as a novel carrier system for both small and large therapeutic molecules. CNTs can be functionalized (i.e., surface engineered) with certain functional groups in order to manipulate their physical or biological properties. In addition to the ability of CNTs to act as carriers for a wide range of therapeutic molecules, their large surface area and possibility to manipulate their surfaces and physical dimensions have been exploited for use in the photothermal destruction of cancer cells. This paper paper will discuss the therapeutic applications of CNTs with a major focus on their applications for the treatment of cancer.

## 1. Introduction

**Figure 1:**
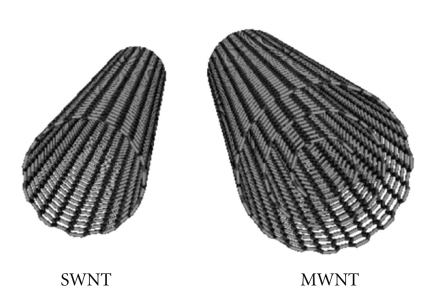
Carbon nanotubes (CNTs) are graphene sheets rolled into a cylindrical shape. Several sheets may roll into MWNTs whilst a single sheet rolls into a SWNT [2].

**Figure 2:**
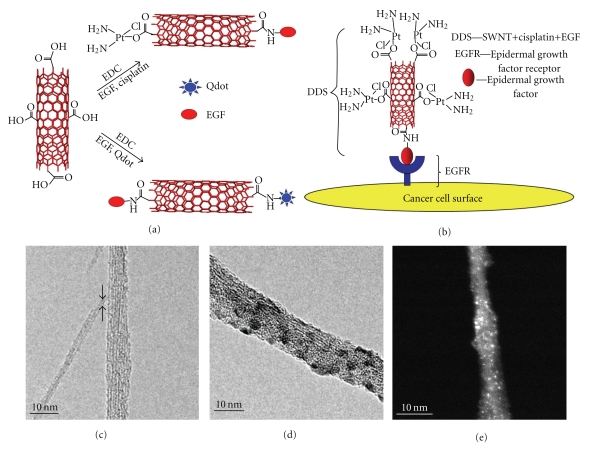
(a) Schematic representation of functionalization of SWNTs with quantum dots, EDC, and cisplatin; (b) SWNT bioconjugated with cisplatin and EGF, targeting cancer cell surface receptor EGFR; (c)–(e) TEM images showing the various functional groups, with cisplatin shown as bright spots [7].

The major aim of developing nanocarrier drug delivery systems is to enhance the therapeutic effect or reduce toxicity of therapeutically active materials. This is conventionally achieved using spherically shaped vesicle nanocarriers such as liposomes. Alternatively, carbon nanotubes (CNTs) are essentially cylindrical molecules made of carbon atoms. CNTs are graphene sheets rolled into a seamless cylinder that can be open ended or capped, having a high aspect ratio with diameters as small as 1 nm and a length of several micrometers. CNTs made from a single graphene sheet results in a single-walled nanotubes (SWNT) while several graphene sheets make up multiwalled carbon nanotubes (MWNTs) [1, 2] (Figure 1). Ever since their discovery in 1991 by Iijima [1], there has been intense interest in these allotropes of carbon due to their unique physical and chemical properties and potential applications in a wide range of fields, from electronic devices and sensors to nanocomposite materials of high strength and low weight. Pristine CNTs are not soluble. It was only after the development of strategies to functionalize these molecules with organic groups and render them soluble that opened the way to bioapplications of CNTs. Due to their high surface area, they are capable of adsorbing or conjugating with a wide variety of therapeutic molecules. Thus, CNTs can be surface engineered (i.e., functionalized) in order to enhance their dispersability in the aqueous phase or to provide the appropriate functional groups that can bind to the desired therapeutic material or the target tissue to elicit a therapeutic effect. CNTs might help the attached therapeutic molecule to penetrate through the target cell to treat diseases [3–6] and a recent example of CNTs with a variety of functional groups relevant to cancer therapy [7] is shown in Figure 2. Here, we provide an overview of the therapeutic applications of CNTs with a major focus on their use in the treatment of cancer. 

## 2. Cellular Uptake of CNTs

**Figure 3:**
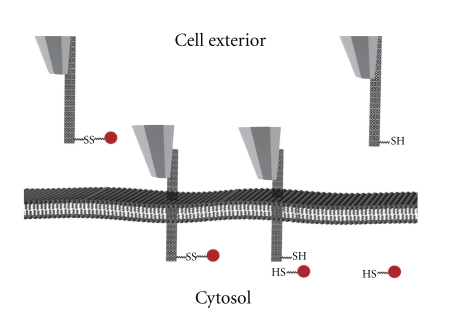
A schematic diagram showing that an AFM-controlled MWNT-based nanoinjector was able to penetrate into a cell and release the attached cargo compound after the breakage of the disulfide bond. This was followed by successful retraction of the nanoinjector with no apparent cell damage being produced [6].

The cellular uptake of CNTs has been confirmed in a range of studies but the mechanism of CNT penetration into cells is still not well understood. Because of their needle-like shapes, CNTs might be able to perforate cellular membrane and pass into the cellular components without causing apparent cell damage [3, 4, 8–11]. An *in vitro* CNTs nanoinjector system has been developed by Chen and coworkers [6]. The nanoinjector was designed using an atomic force microscope (AFM) tip and functionalized MWNTs attached to a model cargo compound via a disulfide linker. The MWNTs nanoinjector successfully transported into the cell where the disulfide bond was broken, resulting in the release of the cargo compound within the cytosol (Figure 3). 

**Figure 4:**
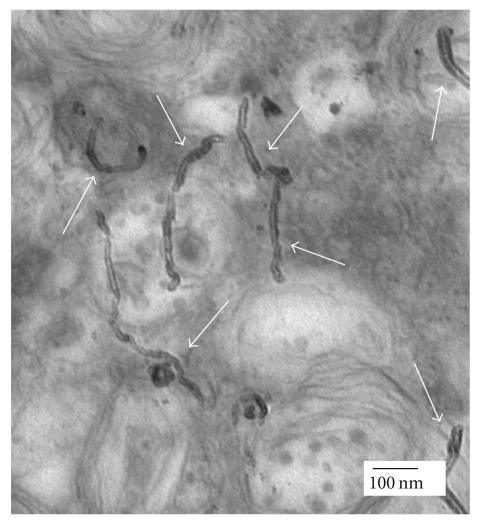
The perpendicular positioning of MWNTs (pointed at by the white arrows) during internalization into HeLa cells suggests that cellular uptake of CNTs by the cells was similar to that of nanoneedles [5].

The perpendicular positioning of the nanotubes to the cell membranes suggests that uptake of CNTs was similar to that of nanoneedles which diffuse through cell membrane without causing cell death [5] (Figure 4). In a study conducted by Kam and coworkers [12], fluoreceinated protein attached to SWNTs-biotin was detected in the endosomes, suggesting that uptake of the nanotubes occurred via endocytosis. By contrast, no protein was internalized in the absence of the nanotubes. Using epifluorescence and confocal microscopy, functionalized CNTs labeled with a fluorescent agent have been shown to penetrate through the cell to the cytoplasm or the nucleus of fibroblasts [3]. In another study, it has been reported that the uptake mechanism of MWNTs is highly dependent on the length of nanotubes since those which are shorter than 1 *μ*m were easier to internalize into cells and the process of cellular uptake was reported not to be via endocytosis [13].

## 3. CNTs as Carriers for Drugs, Genes, and Proteins

CNTs have been investigated as potential nanocarriers for the delivery of drugs, genes, and proteins. Most of the research on CNTs has focused on their potential for delivery of anticancer agents. This might be attributed to their unique needle-like shapes which enable them to be functionalized in order to adsorb or covalently link to a wide variety of therapeutic materials and internalize them into the target cell. Moreover, the well-established safety of vesicle-based carriers particularly liposomes has discouraged many researchers from investigating CNTs in the treatment of many diseases other than cancer. 

### 3.1. CNTs as Carriers of Anticancer Molecules

It is well known that cancer cells overexpress folic acid (FA) receptors, and several research groups have designed nanocarriers with engineered surfaces to which FA derivatives can be attached. Moreover, nonspherical nanocarriers (e.g., CNTs) have been reported to be retained in the lymph nodes for longer periods of time compared to spherical nanocarriers [14] (e.g., liposomes). Thus, CNTs might be used for targeting lymph node cancers as shown by various investigators [15–17]. In these studies, magnetic nanoparticles containing the anticancer cisplatin were entrapped into folic-acid-functionalized MWNTs. An external magnet was employed to drag the nanotubes to the lymph nodes where the drug was shown to be released over several days and the tumor to be selectively inhibited. In a recent study, Yang et al. [18] have loaded the anticancer molecule gemcitabine into magnetic MWNTs and, using mice, they reported high activity against lymph node metastasis when the formulation was injected subcutaneously [18]. In another study, the poorly water-soluble anticancer camptothecin has been loaded into polyvinyl alcohol-functionalized MWNTs and reported to be potentially effective in treatment of breast and skin cancers [19]. 

**Figure 5:**
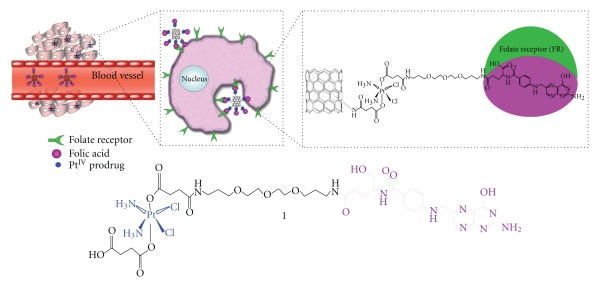
The “longboat” anticancer system in which the chemotherapeutic agent cisplatin is attached from one end to the FA derivative and from the opposite end to a SWNT via an amide link [20].

Dhar and coworkers [20] have developed what they called the “longboat delivery system” (Figure 5). A complex of cisplatin and FA derivative was attached to a functionalized SWNT via a number of amide bonds to comprise the “longboat” which has been reported to be taken up by cancer cells via endocytosis, followed by the release of the drug and its subsequent interaction with the nuclear DNA. Another platinum anticancer, namely, carboplatin, after being incorporated into CNTs has been shown to inhibit the proliferation of urinary bladder cancer cells* in vitro*. In another study, anticancer effects have been shown to be dependent on the method used to entrap the drug in the CNTs, which highlighted the possible effects of preparation conditions on the therapeutic activity of therapeutic molecules associated with CNTs [21]. 

 Paclitaxel is a poorly water-soluble anticancer molecule. In the commercialized paclitaxel product (Taxol), Cremophor EL is used to solubilise the drug. Unfortunately, Cremophor EL itself is toxic, which makes finding a suitable alternative a high priority. Moreover, the circulation time of Taxol is very short. Coating the nanocarriers (e.g., liposomes) with hydrophilic polymers such as polyethylene glycol (PEG) has been established as a strategy to prolong circulation of the nanocarrier-entrapped molecules in the blood by making the carrier highly evasive to uptake by the blood macrophages [22, 23]. PEGylation of paclitaxel increases the circulation time in the blood over Taxol [24]. Functionalized SWNTs were conjugated with paclitaxel through branched PEG chains via a cleavable ester bond. The resultant formulation was more effective in suppressing tumour growth *in vivo* than Taxol or paclitaxel-PEG conjugate in a 4T1 breast cancer animal model. The PEGylated nanotubes were able to prolong the circulation and greatly enhance cellular uptake of the drug by the cancer cells [25]. Similar findings of anticancer activity have been recently shown when paclitaxel was loaded into PEGylated SWNTs or MWNTs using HeLa cells and MCF-7 cancer cell lines [26]. Multidrug resistance is a significant obstacle to successful anticancer drug therapy since the P-glycoprotein efflux transporter can interfere with the accumulation of anticancer drugs in the target cells, resulting in reduced effectiveness of therapy [27, 28]. Recently, using hepatoma cell lines, PEGylated MWNTs have been shown to accumulate in multidrug resistant cells as efficient as in nonresistant cells, as observed by confocal microscopy [29]. Interestingly, Liu and coworkers reported that although PEGylation of SWNTs can prolong blood circulation time of the associated anticancer molecule, it may cause an accumulation of the nanotubes in the dermal tissues of mice, suggesting that the degree of coating with PEG requires optimisation [30]. 

**Figure 6:**
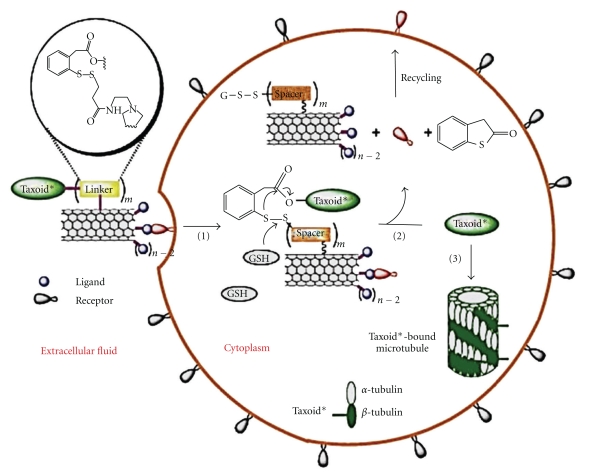
(1) Internalization of the CNTs carried conjugate into the tumour cell via receptor-mediated endocytosis. (2) Taxoid was released by the cleavage of the chemical linker. (3) The free taxoid molecules were bound to microtubules to form stabilized microtubules, resulting in arrest of cell mitosis and induction of apoptosis [31].

Similar to FA receptors, biotin receptors may be overexpressed on the surfaces of certain cancer cells. Therefore, biotin-functionalized SWNTs conjugated with the anticancer agent taxoid using a cleavable linker have been designed [31]. The drug was transported via endocytosis, released in the cell and interacted with microtubules as evaluated by flow cytometry. This resulted in the formation of a stable microtubule-taxoid complex and finally caused apoptosis and cell death (Figure 6).

**Figure 7:**
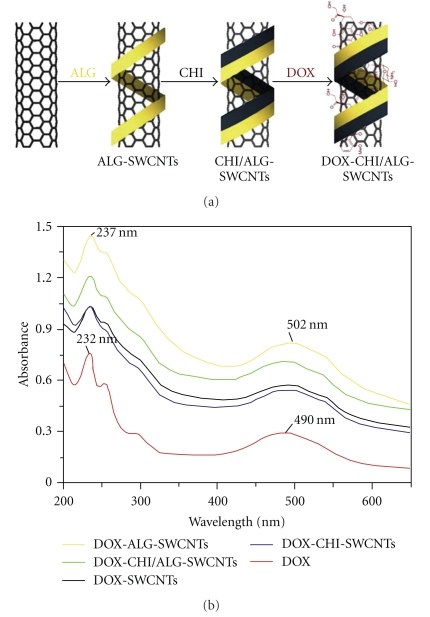
(a) Preparation of SWNTs-DOX after inclusion of bioadhesive polymers to enhance nanotubes dispersability in aqueous phase. (b) UV absorption spectra of DOX formulations [32].

**Figure 8:**
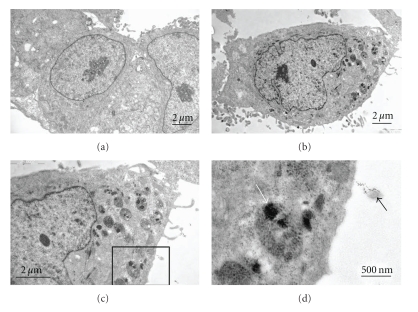
TEM showing the difference between HeLa cancer cells before treatment and after treatment with carbon nanotube formulations and the fate of the nanotubes: (a) HeLa cells before treatment, (b) HeLa cells treated with DOX-FA-CHI-ALG-SWNTs, (c) a magnified image of (b), and (d) magnified image of the boxed region in (c). The black arrow points at a SWNT-containing vesicle, and the white arrow points at some aggregated nanotubes inside a lysosome [32].

A targeted delivery system of FA-tethered SWNTs-doxorubicin (DOX) has been designed. Bioadhesive polymers such as chitosan (CHI) and sodium alginate (ALG) were included to enhance the aqueous dispersability of the nanotubes while FA was used to improve the targeting properties of the nanotubes (Figure 7). Transmission electron microscopy (TEM) indicated that the drug was released after being transported into the tumor cell (HeLa cell) at the low pH of the lysosome [32] but not at the normal physiological pH of 7.4 (Figure 8). Recently, a novel approach has been introduced by covalently attaching PAMAM dendrimers to FA-treated MWNTs. Using this approach to deliver nanotubes, flow cytometry and confocal microscopy indicated possible targeting of cancer cells that overexpress FA receptors [33]. Li and coworkers (2011) have designed a novel system which was referred to as “dual-targeted drug nanocarrier” by conjugating MWNTs with iron nanoparticles and folate molecules. This system was efficiently loaded with doxorubicin and demonstrated superior delivery to HeLa cells when compared to free doxorubicin [34].

### 3.2. CNTs as Carriers of Immunoactive Compounds, Proteins, and Genetic Materials

**Figure 9:**
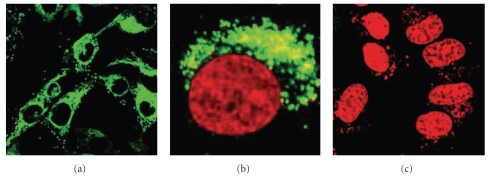
Confocal microscopy showing the internalization of labeled single strand DNA into Hela cell using SWNTs. (a) The labeled DNA (green colour) is surrounding the nucleus (black circles) at 37°C. (b) The nucleus stained using DRAQ5 (red color) is surrounded by the labeled DNA (green colour) after internalization at 37°C. (c) At 4°C, no DNA internalization has occurred [39].

The ability of macromolecules (e.g., genes) to cross the biological barriers and be expressed within a target cell is particularly challenging, owing to their hydrophilicity and large molecular size. Gene therapy aims to use genetic material to treat diseased cells by repairing the cause of the disease. Since genetic materials are poorly able to cross the biological membranes, the use of viral or nonviral vectors to carry the gene and internalize it into the cell is necessary. Nonviral vectors are less efficient than viral vectors [35] and short lived [36]; however, they are far safer [37, 38]. Pantarotto and coworkers have developed novel functionalized SWNT-DNA complexes and reported high DNA expression compared with naked DNA [4]. Generally, functionalized SWNTs have been suggested as suitable nonviral carriers of macromolecules and internalization of such macromolecules into living cells by CNTs has been reported to take place via energy-dependent endocytosis [39]. Confocal microscopy and flow cytometry have shown much greater fluorescent activity of protein and DNA when conjugated to SWNTs as compared to the naked macromolecules [40] indicating that CNTs are promising vectors for gene and protein. Cai and coworkers have introduced an approach to gene delivery named “carbon nanotube spearing” [10]. Plasmid DNA with a fluorescent protein were immobilized onto nickel-embedded CNTs. The formulation was “speared” into Bal 17 B lymphoma cells using a magnetic field, which produced high transfection in the target cells [10]. In another study, exposure of HeLa cells to DNA attached to SWNTs at 37°C resulted in gene internalization. However, at lower temperature (e.g., 4°C), no internalization took place (Figure 9), possibly because gene transport occurs by energy-dependent endocytosis [39]. 

A more recent strategy in gene therapy, namely, gene silencing, involves the use of small interfering RNA (siRNA). This approach has been shown efficient in the treatment of many diseases including various cancers. In cancer, antitumor immunity might be inhibited by suppressors of cytokine signaling 1 (SOCS1). siRNA can be conjugated to phospholipid-functionalized SWNTs using a cleavable disulfide linker, resulting in efficient gene silencing and subsequent death of the targeted cell [9]. In a recent study, amino-functionalized MWNTs-siRNA complexes have shown successful suppression of tumor and prolonged survival in lung tumor of an animal model [41]. Many types of cancer (e.g., leukemia) can overexpress cyclinA(2), and suppression of this material is expected to prevent tumor growth. Functionalized SWNTs have been designed as carrier for siRNA for internalization into K562 cells and subsequent inhibition of the production of cyclinA(2) and treatment of chronic myelogenous leukemia. It has been found that suppression of cyclinA(2) expression using siRNA-CNTs can promote apoptosis in the targeted tumor [42]. Similar findings have been reported, for instance, functionalized SWNTs have been conjugated to telomerase reverse transcriptase (TERT) siRNA. The target gene was successfully silenced, and tumor growth was inhibited *in vitro* using murine tumor cell lines and *in vivo* using a mouse model [43]. In another study, CNTs cationically functionalized with polyethylene imines have been shown capable of complexing with siRNA and generating a silencing activity of up to 30% and cytotoxicity of up to 60% [44]. 

Streptavidin is a protein that has anticancer activity [45]; however, due to its very large molecular weight (approximately 60,000 Da), it does not penetrate through cells. Kam and coworkers [12] have used a conjugate of streptavidin with SWNTs-biotin, which resulted in internalization of the protein into model cancer cells by adsorption-mediated endocytosis. Transmission electron and confocal microscopy have shown that MWNTs can act as transporters of the recombinant ricin A chain protein, resulting in high death rates of cancer cells (e.g., up to 75% of HeLa cells were killed) [46].

Immunotherapy may be an alternative to gene therapy in the treatment of cancer. Antitumor immunotherapy using CNTs has been recently researched. Tumor-specific monoclonal antibodies, radiometal ion chelates, and fluorescent probe have been attached to SWNTs. Targeting the tumor (lymphoma) using a range of techniques has been reported to be successful [47]. MWNTs have been conjugated to tumour lysate protein as an antigen. This specifically increased the antitumor immune response [48]. 

Glioma is a brain tumor that is able to evade the host immune system, resulting in lack of benefit from conventional chemotherapy. This is because glioma cells secrete the immunosuppressive cytokines such as prostaglandins E and TGF-Beta and IL-10 [17, 49]. Macrophages have a preferential affinity towards CNTs when compared to glioma cells [50]. Using a GL261 murine intracranial glioma cancer model, VanHandel and coworkers [51] have developed an immunotherapy approach using MWNTs based on the fact that macrophages prefer to engulf CNTs compared with glioma cells. MWNTs caused an increase in the influx of macrophages into the glioma cells. This was reported to be accompanied by an increase in the levels of IL-10 expression, suggesting that immunomodulation using CNTs is a possible strategy to treat cancer. 

Angiogenesis targeting antibodies E4G10 were attached to SWNTs via radiometal ion chelates. This formulation has been reported to reduce the volume of the tumor and prolong survival in animal models [52]. Moreover, oxidized MWNTs can be injected subcutaneously to a hepatocarcinoma-bearing animal to induce an immune response, which has been reported to retard tumor growth [53]. This suggests that CNTs themselves could possibly be surface-engineered to have anticancer activity by inducing an immune response against tumor. 

A major obstacle to effective anticancer therapy is the multidrug resistance caused by enhanced efflux of anticancer drugs by the overexpressed p-glycoprotein, resulting in poor anticancer effect. Li and coworkers (2010) have shown that SWNTs can be functionalized with p-glycoprotein antibodies and loaded with the anticancer agent doxorubicin. Compared with free doxorubicin, this formulation demonstrated higher cytotoxicity by 2.4-fold against K562R leukemia cells [54].

### 3.3. Photothermal Therapy of Cancer Using CNTs

CNTs are able to absorb light in the near infrared (NIR) region, resulting in heating of the nanotubes [55]. This unique property of CNTs has been exploited as a method to kill cancer cells via thermal effects [39, 56–68]. 

**Figure 10:**
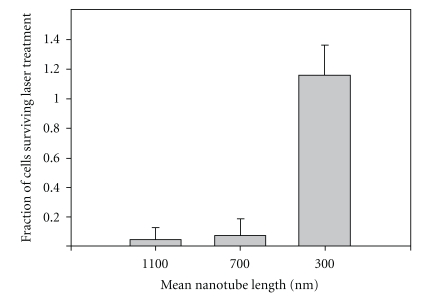
The relationship between cell survival and CNTs length using photothermal therapy. This has shown that nanotube lengths of 700 and 1100 nm are much more desirable in killing tumor cells compared with the length of 300 nm [57].

Optical coupling of light with CNTs is predicted to be at highest when the length of the nanotubes is more than half the wave length of the incident light beam as determined by the antenna theory [66]. Engineering the structure of MWNTs by creating intentional surface defects might enhance the antenna properties of the nanotubes. Such engineered “defects” or dopants will cause scattering in the travelling currents and also increase the heating of the nanotube. This physicoelectronic characteristic of the engineered MWNTs can be employed to thermally destruct the tumor cells by using MWNTs that have good heat conducting properties. Examples of dopants include boron [67] and nitrogen [57] (N-doping). N-doped MWNTs have been shown to produce photoablative kill of model kidney cancer cells when NIR light was used. Moreover, the length of nanotubes has been found to be a major determinant of nanotube ability to transfer heat and kill the tumor with lengths between 700 and 1,100 nm being most desirable to kill the tumor [57] (Figure 10). 

 In a study conducted by Gannon and coworkers [56], SWNTs were functionalized using Kentera (a polyphenylene ethynylene-based polymer). The incubation of the nanotubes with hepatic tumor cells followed by application of radiofrequency field caused a concentration-dependent thermal destruction of the tumor cells which was demonstrated by development of apoptotic cells that caused complete necrosis of the tumor cells. By contrast, tumor cells that were injected with the Kentera alone (without CNTs) were viable after the application of the radiofrequency field. In the same study, it has been reported that *in vivo* injection of the Kentera-functionalized SWNTs was tolerated by rabbits [56]. Unfortunately, the resultant thermal destruction is not selective towards cancer cells and the access to deep tumor areas is generally poor, necessitating the inclusion of targeting moieties such as FA on the surfaces of CNTs [37]. Folate-bearing nanotubes having the size of 0.81 nm and a maximum absorbance at 980 nm were used for photothermal therapy of cancer [58]. The tumor cells were exposed to 980 nm laser radiations, resulting in photothermal destruction of cancer cells both *in vitro* and *in vivo*.

### 3.4. CNTs for Other Therapeutic Applications

The use of CNTs has been expanding to include therapeutic applications other than cancer. For instance, surface-engineered CNTs may be able to capture pathogenic bacteria in liquid medium [69–71]. Thus, CNTs themselves might have antimicrobial activity since microorganisms may be adsorbed onto the engineered surfaces of CNTs. Moreover, using E. coli as a model microorganism, it has been reported that the electronic properties of SWNTs may regulate their antibacterial activity. The antibacterial effect was attributed to carbon-nanotube-induced oxidation of the intracellular antioxidant glutathione, resulting in increased oxidative stress on the bacterial cells and eventual death [72]. 

Functionalized CNTs have been demonstrated to be able to act as carriers for antimicrobial agents such as the antifungal amphotericin B [73, 74]. CNTs can attach covalently to amphotericin B and transport it into mammalian cells. This reduced the antifungal toxicity as compared to the toxicity of the free drug since 40% of the cells were killed by the CNTs-free formulation compared to no cell death by the CNTs formulation. It has also been reported that the antifungal activity was increased using the CNTs [73].

## 4. Conclusions

CNTs are promising needle-like carriers of both small drug molecules as well as macromolecules such as genes and proteins. CNTs can be functionalized so that certain molecules are attached to their surfaces via covalent or noncovalent bonding. The needle-like shape of the CNTs enables them to perforate cellular membranes and transport the carried therapeutic molecules to the cellular components. This process is thought to take place via endocytosis. CNTs have exclusive properties that would make them appropriate in the medical field such as their ability to adsorb pathogenic microorganisms and conduct heat. CNTs have been introduced to drug delivery research for a limited number of years and therefore extensive amounts of research is expected to be produced in the forthcoming years in order to explore their potential.
